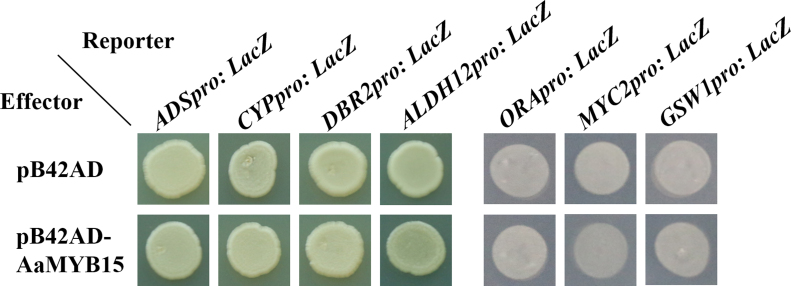# AaMYB15, an R2R3-MYB TF in *Artemisia annua*, acts as a negative regulator of artemisinin biosynthesis

**DOI:** 10.1016/j.plantsci.2021.110920

**Published:** 2021-07

**Authors:** Zhangkuanyu Wu, Ling Li, Hang Liu, Xin Yan, Yanan Ma, Yongpeng Li, Tiantian Chen, Chen Wang, Lihui Xie, Xiaolong Hao, Sadaf-llyas Kayani, Kexuan Tang

**Affiliations:** Joint International Research Laboratory of Metabolic and Developmental Sciences, Key Laboratory of Urban Agriculture (South) Ministry of Agriculture, Plant Biotechnology Research Center, Fudan-SJTU-Nottingham Plant Biotechnology R&D Center, School of Agriculture and Biology, Shanghai Jiao Tong University, Shanghai, 200240, China

**Keywords:** Artemisinin, Negative regulator, *Artemisia annua*, AaMYB15

## Abstract

Artemisinin is a secondary metabolite extracted from *Artemisia annua*. As an effective antimalarial component certified by WHO, artemisinin has extensive economical values. Numerous studies about transcription factors positively regulating artemisinin biosynthesis have been published while negative regulators are rarely reported. In the present study, we identified AaMYB15 as the first R2R3-MYB that negatively regulates artemisinin biosynthesis in *A. annua*. Experimental evidences showed that AaMYB15 is a transcription factor within nucleus and predominantly expressed in glandular secretory trichomes (GSTs) in *A. annua* where artemisinin is synthesized and accumulated. The expression of *AaMYB15* was induced by dark and JA treatment. Overexpression of *AaMYB15* led to a significant decline in the expression levels of key enzyme genes *ADS*, *CYP*, *DBR2*, and *ALDH1* and a significant decrease in the artemisinin contents of transgenic *A. annua*. AaMYB15 directly bound to the promoter of *AaORA*, a reported positive regulator of artemisinin biosynthesis in JA signaling pathway, to repress its transcriptional activity, thus downregulating the expression levels of downstream key enzyme genes and negatively regulating the artemisinin biosynthesis. Our study provides candidate gene for improvement of *A. annua* germplasm and new insights into the artemisinin biosynthesis regulation network mediated by light and JA.

## Highlights

• AaMYB15 negatively regulates artemisinin biosynthesis in *Artemisia annua.*
• AaMYB15 inhibits the promoter activities of *ADS*, *CYP71AV1*, *DBR2*, and *ALDH1* indirectly.
• AaMYB15 binds to *proAaORA* and suppresses its activity, thus downregulating downstream key enzyme genes.
• AaMYB15 responds to dark and JA treatment.

## 1 Introduction

*Artemisia annua* is a traditional Chinese medicinal plant, known as the natural plant source of artemisinin, an antimalarial effective secondary metabolite. Artemisinin-based combination therapies (ACTs) are universally popularized by WHO as a commonly accepted effective method for malaria treatment [1]. Additionally, artemisinin and its derivatives have also been reported to have the potential of anti-antimicrobial [2], antiviral [3], anti-tumor [4], treatment of diabetic [5], treatment of HIV [6] and treatment of lupus erythematosus-related nephritis [7]. Nonetheless, the content of artemisinin in wildtype *A. annua* plants is very low, and the mass fraction can only account for 0.01 %–1 % of the dry weight of the whole plant [8]. The contradiction between demand and supply of artemisinin contributes to the development of yeast semi-synthetic artemisinin precursors [9,10].However, the high cost limits the commercialization of this method. *A. annua* plants are still main source of artemisinin, and study on how to increase artemisinin content in wild plants remains a hotspot of research.

The location of artemisinin biosynthesis and accumulation is revealed to be in the glandular secretory trichomes (GSTs) of *A. annua* [11], and there are multiple key rate-limiting enzymes in the pathway of artemisinin biosynthesis, including ADS, CYP71AV1, DBR2, and ALDH1 [[12], [13], [14], [15]]. According to the characteristics of artemisinin biosynthesis, there have been many strategies to increase artemisinin content so far. Among these strategies, transcriptional regulation of artemisinin biosynthesis through transcription factors is a highly valuable genetic engineering method [16]. Numbers of transcription factor families have been reported to play essential roles in artemisinin biosynthesis formerly, including AP2/ERF family, bHLH family, bZIP family, HD-ZIP family, WRKY family [17], HD-ZIP family [18,19], NAC family [20], TCP family [21], MYB family [[22], [23], [24]] and so forth. However, the vast majority of these transcription factors mentioned above are reported to regulate artemisinin biosynthesis positively, while studies on negative regulators are still quite rare. Study on negative regulators is of great significance to explore the mystery of dynamic balance mechanism in secondary metabolism and to describe the regulatory network more clearly.

MYB TFs are the largest family of plant transcription factors and plays disparate roles in various physiological activities of plants. R2R3-MYB class is the largest in 4 classes of MYB TFs, accounting for 63.6 % of the MYB genes in *Arabidopsis thaliana* [[25], [26], [27]]. Numerous previous research findings have proved that MYB TFs function as positive or negative regulators of secondary metabolism. AtMYB11/PFG1, AtMYB12/PFG1 and AtMYB111/PFG3 were reported to positively regulate multiple flavonoid synthase genes in all tissues of *A. thaliana* [28]. In *Antirrhinum majus* L., AmMYB305 and AmMYB340 were found to participate in promoting biosynthesis of phenylpropane and flavonoids [29]. As for negative regulation, AtMYB68 was reported to negatively regulate tannin biosynthesis in *A. thaliana* [30]. MtMYB2 was shown to negatively regulate the biosynthesis of proanthocyandin and anthocyanin pigmentation in *Medicago truncatula* [31]. However, there have been few reports on R2R3-MYB TFs negatively regulating artemisinin biosynthesis.

Light signaling contributes to regulating various physiological activities of plants, as well as the artemisinin biosynthesis and its accumulation [32]. Earlier studies have suggested that UV-B [33], white light [34] and blue light [35] are relevant to the synthesis of artemisinin. Experimental evidences have implied that small amount of UV-B treatment significantly promotes the expression of artemisinin biosynthesis related genes and increases the content of artemisinin [33]. Cool-white light treatment has been confirmed to increase the growth and artemisinin production in the hairy roots of *A. annua* [34]. Moreover, overexpression of blue light receptor *AtCRY1* in *A. annua* has been reported to activate the expression of artemisinin biosynthesis key enzyme genes *AaADS* and *AaCYP71AV1*, thereby increasing the artemisinin content in transgenic plants by 30–40 % [35]. Expression levels of artemisinin biosynthesis genes in dark condition are also found to be significantly inhibited in comparison with those in light condition [36]. Jasmonates (JA) signaling is also identified as a regulator of artemisinin biosynthesis. Experimental evidences show that exogenous JA treatment increase the GSTs density of *A. annua*. JA signaling also regulates artemisinin biosynthesis by activating TFs which regulate GSTs density or expression levels of related genes, including AaORA [37], AaHD1 [18], AaMYC2 [38], AaGSW1 [39], AaERF1 and AaERF2 [40]. Among these TFs, AaORA was reported as a trichome-specific transcription factor from AP2/ERF family, acting as a crucial positive regulator in JA-mediated signaling pathway. It was found to positively regulate the expression levels of key enzyme genes *ADS*, *CYP71AV1*, and *DBR2* significantly [37], thus increasing the content of artemisinin in *AaORA* overexpression transgenic plants. In other secondary metabolic regulation pathway, there were reports of JA-induced negative regulators [41], implying that there was a subtle balance mechanism in JA-mediated regulation. Besides, previous research of our group revealed that the regulation on artemisinin biosynthesis of JA signaling depended on presence of light and transcriptome sequencing analysis was performed to further elucidate the specific mechanism [36].

*AaMYB15* was selected as a differentially expressed gene (DEG) between light, dark and JA treatment from transcriptome analysis mentioned above [36] and was identified as a R2R3-MYB gene. In this study, expression of *AaMYB15* was found to be inhibited in light condition and induced by dark and JA treatment. In addition, AaMYB15 directly binds to the promoter of *AaORA* and represses its activity to downregulate the expression of multiple key enzyme genes in artemisinin synthesis pathway indirectly. In general, our study reveals that AaMYB15 is a negative regulator of artemisinin biosynthesis and provides a puzzle piece for the network of regulation mediated by light signaling and JA signaling.

## 2 Materials and methods

### 2.1 Plant materials and growth conditions

*A. annua* seeds used for stable or transient transformation and other *A. annua* related assay in this study was a high-artemisinin cultivar named “Huhao 1”, which was originated from Chongqing and further developed in Shanghai for years of selection [38]. *Nicotiana benthamiana* seeds were used for transient transformation. All seedlings were planted in pots filled with vermiculite and peat at 25℃.

### 2.2 Bioinformatics analyses of AaMYB15

Sequences of *AtMYB* genes were obtained from TAIR (https://www.arabidopsis.org/) database and phylogenetic tree was constructed with MEGA X (https://www.megasoftware.net/) by Neighbor-Joining method. Hierarchical Cluster heatmap based on previous transcriptome analysis was illustrated with MeV (https://sourceforge.net/projects/mev-tm4/files/mev-tm4/), and IDs of AaMYB TFs involved were retrieved from *A. annua* proteome data (NCBI ID：txid35608) with HMMER-3.0 (http://www.hmmer.org/) and hidden Markov model of MYB family downloaded from Pfam (http://pfam.xfam.org/) database. MYB protein sequences with high homology from other species were downloaded from NCBI (https://www.ncbi.nlm.nih.gov/) database. Amino acid sequence alignment was performed by ClustaW tool in MEGA X software and GeneDoc (http://www.flu.org.cn/en/download-47.html).

### 2.3 Treatment of light, dark and JA

For light and dark treatment, 14-day-old *A. annua* seedlings with same growth status were selected for subsequent use. Half (10 pots, 4 plants per pot) were kept under continuous illumination and half (10 pots) transferred to completely dark conditions. Leaves were sampled at 0, 0.5, 1, 3, 9, 12 and 24 h during treatment, and frozen in liquid nitrogen for RNA extraction.

For JA treatment, 14-day-old *A. annua* seedlings with same growth status were treated with MeJA of 100 μmol/L, then sampled at 0, 0.5, 1, 3, 9, 12, and 24 h after treatment.

### 2.4 RNA extraction and quantitative real-time-PCR (qRT–PCR)

For analysis of expression patterns of *AaMYB15* in different tissues and various leaf positions, samples of wildtype *A. annua* were gathered from different tissues (root, stem, young leaf, old leaf, buds, flower, shoot and trichome) and leaves (the apical meristem leaves and the 1 st, 2nd, 3rd, 5th, 7th, 9th, and 12th positions below). Samples of transgenic *A. annua* were collected from the first lateral branch below the apical meristem of each seedling. All samples were frozen in liquid nitrogen and total RNA was extracted with OminiPlant RNA Kit (CW Biotech, China). The extraction procedure was based on the kit instructions, and concentrations were measured by Nanodrop. cDNA was obtained by reverse transcription with PrimeScript 1 st Strand cDNA Synthesis Kit (Takara, Japan) according to the instructions.

QRT–PCR analyses were performed with TB Green® Premix EX Taq™ II (Takara, Japan). Three technical and biological repeats were replicated for each analysis. *β-ACTIN* was set as a standard/ control. All primers related were listed in Table S1.

### 2.5 Subcellular localization assays

Full-length coding sequence of *AaMYB15* was inserted into pHB-YFP vector to generate pHB-AaMYB15-YFP. The plasmid was transferred into *Agrobacterium tumefaciens* (strain GV3101). After overnight culturing and OD_600_ adjusted to 600 with MS_0_ liquid medium, GV3101 harboring pHB-AaMYB15-YFP was injected into subfoliate epidermis of *N. benthamiana* (about 4-week-old) with 1 mL needleless syringes for transient transformation. Seedlings were kept under dim light for 48 h at 25℃ to complete infection and gene expression. The YFP signal was observed under confocal laser microscope (Leica TCS SP5-II, Leica Microsystems, Wetzlar, Germany) at 530−600 nm. DAPI (Sigma-Aldrich, USA) was used to stain nuclei and the infiltration was performed 1 h before observation.

### 2.6 Overexpression and antisense vectors construction and transformation of *A. annua*

To overexpress or silence *AaMYB15* gene, forward and reverse complementary coding sequences of *AaMYB15* were cloned and inserted into pHB-YFP driven by the CaMV35S promoter to generate pHB-AaMYB15-YFP and pHB -AaMYB15-antisense constructs.

Sterilized seeds of *A. annua* were sown on MS_0_ plates and cultured at 25℃ until seeds germinate and seedlings grow to 4−5 cm.The plasmids mentioned above were transferred into *A. tumefaciens* (strain EHA105) used to infect *A. annua* seedlings. Leaves were cut and soaked with EHA105 strains on co-cultivation medium in dark condition for 3 days, then transferred to germination screening medium MS_1_ and root induction medium MS_2_, respectively. Afterwards, screened *A. annua* seedlings were transplanted into pots filled with vermiculite and peat for growth. Transgenic *A. annua* seedlings were confirmed after DNA extraction and positive detection by PCR. Detailed formulations of MS_0_, MS_1_ and MS_2_ mentioned above referred to our previous research [42]. All primers related were listed in Table S1.

### 2.7 Dual-Luciferase assay

The promoter regions of *ADS, CYP71AV1, DBR2, ALDH1,* and *AaORA* were cloned and inserted into pGREENII0900-LUC vector(reporters). Coding sequence of *AaMYB15* was inserted into pHB-YFP vector and empty pHB-YFP was used as negative control (effectors). Effector plasmids and reporter plasmids were severally transferred into GV3101 strains and GV3101 strains with helper plasmid pSoup19, respectively. Strains harboring effectors and reporters were 1:1 mixed and co-transferred into subfoliate epidermis of *N. benthamiana*. Plants were kept in low light condition at 25℃ for 48 h to allow infection and genes expression. Samples were collected into 1.5 mL centrifuge tubes with 2 steel beads and frozen in liquid nitrogen, then ground by a high throughput tissue grinder at 55 Hz for 20 s twice. Firefly LUC and REN activities were detected and analyzed by GloMax 20/20 Luminometer and Dual-Luciferase® Reporter Assay System Kit (Promega, USA), with procedure according to instructions of the kit.

### 2.8 GUS expression assay by transient transformation of *A. annua*

The 1630-bp promoter region upstream of the initiation codon of *AaMYB15* was cloned and inserted into pCambia1391Z vector with GUS reporter to construct 1391Z-proAaMYB15-GUS. Afterwards, the plasmid was transferred into GV3101 strains in preparation for the transient transformation of *A. annua* seedlings.

GV3101 strains were injected into young leaves of 2-week-old *A. annua* seedlings with needleless syringes. After 48 h cultivation in low light condition, those seedlings were soaked into GUS staining solution at 37℃ overnight in the dark to be stained. Subsequently, anhydrous ethanol was used to remove chlorophyll at 65℃, and GUS staining activity was observed and recorded under an optical microscope.

### 2.9 Yeast one-hybrid (Y1H) assay

Full-length coding sequence of *AaMYB15* was cloned and inserted into pB42AD vector to construct pB42AD-*AaMYB15*. Analysis of *AaORA* promoter indicated that there are MYB-binding motifs in the promoter region. Whole promoters of *ADS*, *CYP71AV1*, *DBR2*, *ALDH1*, *AaORA*, *AaMYC2*, *AaGSW1*, and three tandem repeats of a motif *M6* were ligated into pLacZ vector. Empty pB42AD and pLacZ vectors were used as negative controls. Various combinations of plasmids were co-transferred into yeast strain EGY48A. Strains harboring different combinations of plasmids were cultured on SD/-Trp/-Ura plates and interaction status were tested on qualitative SD/-Trp/-Ura medium plates containing X-gal by observing whether the colonies turn blue.

### 2.10 Yeast two-hybrid (Y2H) assay

Full-length coding sequence of *AaMYB15* was cloned and inserted into pGADT7 vector and other genes including *AaGSW1*, *AaHD1*, *AaERF1*, *AaJAZ8*, *AabHLH1*, *AabZIP1*, *AaHY5*, *AaCOP1* were inserted into pGBKT7 vector. Empty pGADT7 and pGBKT7 vectors were used as negative controls. Various combinations of plasmids were co-transferred into yeast strain AH109 and transformed strains were cultured on SD/-Trp/-Leu plates. Interaction status were tested on SD/-Trp/-Leu (DDO), SD/-Trp/-Leu/-His (TDO) and SD/-Trp/-Leu/-His/-Ade (QDO).

### 2.11 Electrophoretic mobility shift assays (EMSA)

Full-length coding sequence of AaMYB15 was cloned and inserted into pCold -TF vector (Takara, Japan) for protein purification. Empty pCold -TF vector was used as negative control. The plasmids were transferred into *E. coli* strain Rosetta (DE3) (TransGen Biotech, China). HisSep Ni-NTA Agarose Resin and 5 mL Gravity Chromatography Colimns (Yeasen, China) were used to purify fusion proteins.

EMSA was performed according to instructions of LightShiftTM EMSA Optimization& Control Kit (Thermo Fisher Scientific, China). Biotin-labeled probes of ORAM6 and ORAM6-mutated and unlabeled probes as competitor were synthesized by Sangon, China. All primers and probes related were listed in Table S1.

### 2.12 Measurement of artemisinin content

pHB-AaMYB15-YFP (OE-AaMYB15) and pHB-AaMYB15-antisense **(**AN-AaMYB15) transgenic *A. annua* seedlings were cultivated at 25 ℃ for 3 months. Subsequently, samples were collected from leaves and dried in a thermostatic blast dryer at 50℃.Dry leaves samples were ground to powder by a high-throughput tissue grinder at 55 Hz for 30 s and 0.1 g powder was weighed by analytical balance from each sample. Artemisinin was extracted by 2 mL (repeat twice, 1 mL each time) under ultrasound for 30 min at 30 ℃ and filtered by 0.22 μm nitrocellulose filters. Concentrations were determined by HPLC-ELSD with Waters Alliance 2695 HPLC system and a Waters 2420 ELSD detector (Milford, USA) The HPLC and ELSD conditions were as described previously [37]. Artemisinin used as standard was purchased from Sigma-Aldrich, USA.

## 3 Results

### 3.1 Identification of AaMYB15

**Fig. 1:**
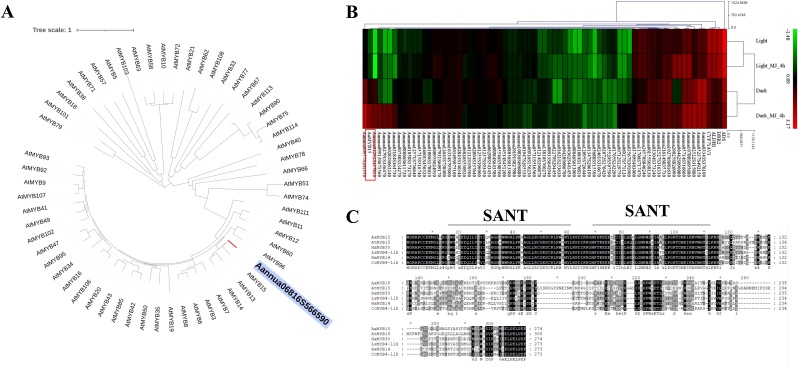
Identification of AaMYB15. (A) Phylogenetic tree constructed with neighbor-joining method, showing the relationship between AaMYB15 and MYB TFs sequences of Arabidopsis thaliana. Line length represents evolutionary distance; Each node number represents bootstrap value (repeated 1000 times). This bootstrapped tree with 1000 replicates was made using ClustalW tool in MEGA 6.0. (B) Hierarchical cluster analysis of MYB transcription factor in A. annua. The color scale on the right represents the reads from millions of mappings per thousand bases. Data is processed by log10. The red box marks the candidate genes. (C) Alignment of AaMYB15 and sequences from *A. thaliana* (AT3G23250.1), *Helianthus annuus* (XP_022002601.1 and XP_022029957.1), *Lactuca sativa* (XP_023735423.1) and *Cynara cardunculus* var. scolymus (XP_024996317.1). The two conserved MYB domains are marked by lines above sequences.

Light, dark and JA treatment transcriptome analysis was completed in our previous study [36], in which Aannua06816S566590 was identified as a conspicuous DEG. The length of opening reading frame (ORF) was 825bp, encoding a predicted R2R3-MYB TF with 274 amino acids. Phylogenetic tree analysis was performed between Aannua06816S566590 and 59 reported R2R3-MYB TFs in *Arabidopsis thaliana*. The result demonstrated that Aannua06816S566590 had the highest homology with AtMYB15 (Fig. 1, A).

Based on transcriptome sequencing data of *A. annua* treated by light, dark and JA, Hierarchical Cluster Heatmap of ADS, CYP71AV1, DBR2, ALDH1 and 70 MYB TFs in *A. annua* including Aannua06816S566590, designated AaMYB15, was illustrated (Fig. 1, B). The results of cluster analysis showed that the expression of *AaMYB15* was inhibited when light existed and highly induced by dark and JA treatment, which was contrary to the expression patterns of key enzyme genes and typical positive regulators of artemisinin biosynthesis. Combined with GSTs transcriptome sequencing data maintained by our research group, AaMYB15 was found to express dominantly in GSTs, indicating that it might be relevant to artemisinin synthesis and accumulation.

Analysis of protein sequences among AaMYB15, AtMYB15 and several high homologous genes in other plants from Compositae (Fig.1, C) showed that AaMYB15 contained two typical conserved MYB repeat domains for DNA binding and had close relationship with R2R3-MYB TFs in other Compositae plants.

Taken together, the R2R3-MYB TF, AaMYB15 was selected as the candidate gene of this study, assumed to be a negative regulator of artemisinin biosynthesis and involved in light and JA signaling pathways.

### 3.2 Expression patterns of AaMYB15

**Fig. 2:**
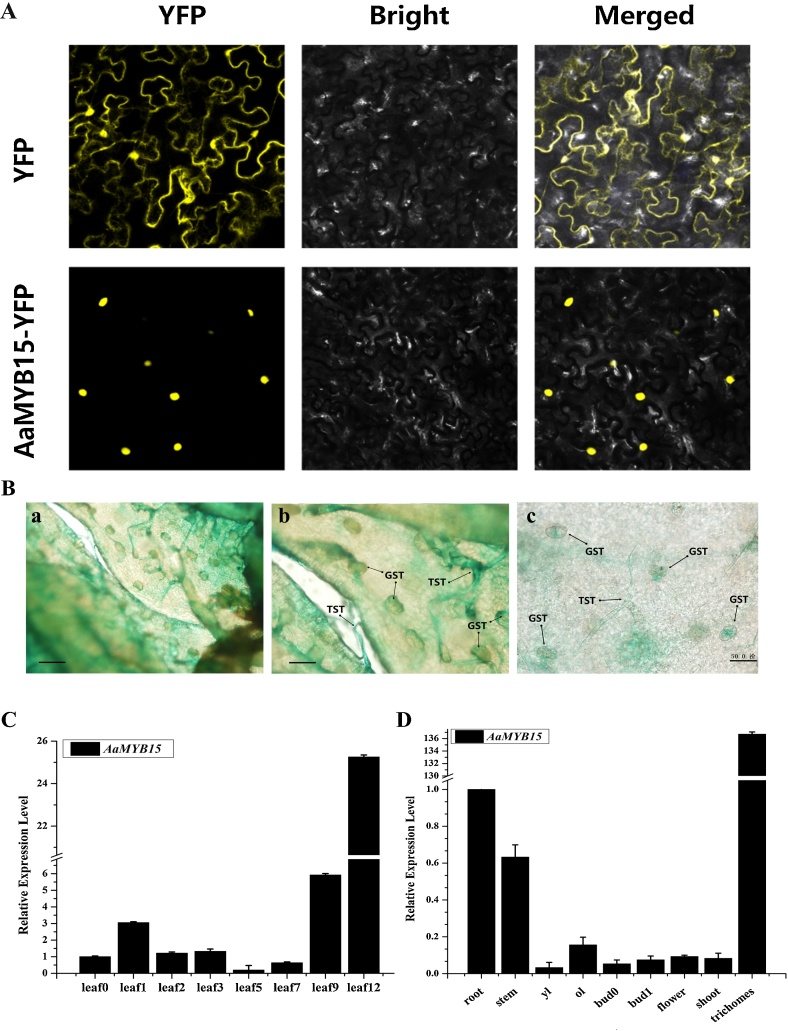
Expression patterns of *AaMYB15.* (A) Subcellular localization of AaMYB15.The upper row is 35S：YFP as the positive control, while the lower row is 35S:AaMYB15-YFP and the yellow was the YFP fluorescence. (B) Fig. 3, Fig. 4, Fig. 5 GUS-staining pattern of 1391Z-proAaMYB15-GUS.GSTs and TSTs are marked by black arrows in these pictures. B is the enlarged view of A. Scale bars were 200 μm (a) and 50 μm (b and c). (C) Relative expression levels of *AaMYB15* measured by qRT-PCR in leaves at different positions. Values are given as means ± SD from three biological replicates. (D) Relative expression levels of *AaMYB15* measured by qRT-PCR in different tissues. Values are given as means ± SD from three biological replicates.

To explore the location where the AaMYB15 protein functions within cells, AaMYB15-YFP fusion protein was constructed and transiently transformed into *N. benthamiana*. AaMYB15-YFP was observed to be localized in the nucleus specifically, whereas empty YFP as the control, was found distributed throughout the cell (Fig. 2, A). Nuclei were stained with DAPI (Fig. S1). In order to further determine the expression pattern of *AaMYB15*, 1391Z-proAaMYB15-GUS, which was constructed with the 1630-bp promoter region upstream of the initiation codon of *AaMYB15* and pCambia1391Z vector with GUS reporter, was transiently transformed into wildtype *A. annua*. Gus staining was recognized in GSTs and T-shape trichomes (TSTs) (Fig. 2, B), showing GUS activity and indicating that *AaMYB15* was expressed in GSTs and TSTs. To investigate the spatial and temporal expression patterns of AaMYB15, qRT-PCR analyses were performed to detect expression level of *AaMYB15* in leaves at different positions and different tissues as well (Fig. 2, C and D). It was revealed that the expression level was gradually increased during the development of leaves and predominant in trichomes.

These results indicate that AaMYB15 is a potential transcriptional regulator that functions in the nucleus and most likely to be associated with artemisinin biosynthesis.

### 3.3 *AaMYB15* is induced by dark and JA treatment

**Fig. 3:**
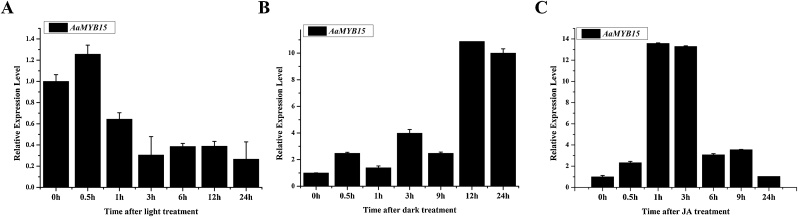
AaMYB15 is induced by dark and JA treatment. (A) Relative expression levels of *AaMYB15* measured by qRT-PCR under light treatment. Values are given as means ± SD from three biological replicates. (B) Relative expression levels of *AaMYB15* measured by qRT-PCR under dark treatment. Values are given as means ± SD from three biological replicates. (C) Relative expression levels of *AaMYB15* measured by qRT-PCR under JA treatment. Values are given as means ± SD from 3 biological replicates.

To reconfirm how AaMYB15 respond to light, dark and JA treatment, expression levels of *AaMYB15* under light, dark and JA treatment were detected by qRT-PCR in treated wildtype *A. annua* seedlings (Fig. 3, A–C). The expression level was significantly upregulated in the dark and reached a peak at 12 h after the treatment, while downregulated in the light. When exposed to JA treatment, the expression level of *AaMYB15* increased rapidly and reached a peak at 1 h after the treatment. These results are consistent with the transcriptome analysis that AaMYB15 is a transcriptional factor induced by darkness and JA, implying that it might participate in light and JA-mediated regulation of artemisinin biosynthesis.

### 3.4 AaMYB15 negatively regulates artemisinin biosynthesis in transgenic *A. annua*

**Fig. 4:**
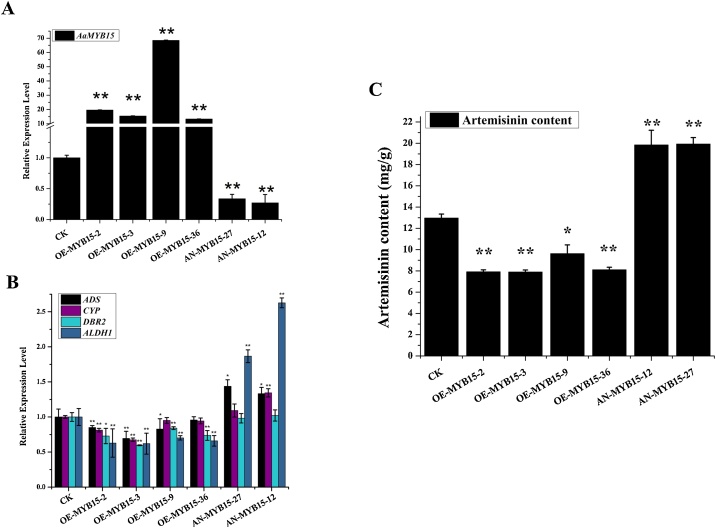
AaMYB15 negatively regulates artemisinin biosynthesis in transgenic *A. annua*. (A) Expression levels of *AaMYB15* in overexpression of *AaMYB15*(OE-MYB15) and antisense-AaMYB15(AN-MYB15) transgenic A. annua. Values are given as means ± SD from three biological replicates. Asterisks indicate significant differences between transgenic plants and CK by Student’s *t*-test. (**, P < 0.01；*,P < 0.05.) (B) Expression levels of *ADS*, *CYP71AV1*, *DBR2* and *ALDH1* in transgenic plants. Values are given as means ± SD from three biological replicates. Asterisks indicate significant differences between transgenic plants and CK by Student’s *t*-test. (**, P < 0.01；*,P < 0.05.)(C) Artemisinin content in overexpression of *AaMYB15*(OE-MYB15) and antisense-*AaMYB15*(AN-MYB15) transgenic A. annua. measured by HPLC-ELSD. Values are given as means ± SD from three biological replicates. Asterisks indicate significant differences between transgenic plants and CK by Student’s *t*-test. (**, P < 0.01；*,P < 0.05.)

In order to determine how AaMYB15 functioned in artemisinin biosynthesis in *A. annua*, two genotypes of stable transgenic *A. annua* lines, including overexpression of *AaMYB15*(OE-MYB15) and antisense-*AaMYB15*(AN-MYB15) driven by the CaMV35S promoter, were obtained to analyze effects of AaMYB15 on artemisinin biosynthesis in *A. annua*. In order to confirm the effect of transformation, qRT-PCR was performed to detect expression level of *AaMYB15* in transgenic plants (Fig. 4, A). It was shown that expression of *AaMYB15* was increased in OE-MYB15 plants and decreased in AN-MYB15 plants. Expression levels of *ADS, CYP71AV1, DBR2,* and *ALDH1* were checked by qRT-PCR analysis (Fig. 4, B). The data suggested that transcriptional activities of the enzymes were inhibited in OE-MYB15 plants and enhanced in AN-MYB15 plants in comparison with wildtype control check (CK). Additionally, values of the artemisinin content in transgenic *A. annua*. seedlings were measured by HPLC-ELSD (Fig. 4, C). As the results suggested, the artemisinin concentrations in OE-MYB15 plants were significantly declined compared to CK while those in AN-MYB15 plants were substantially increased.

These experimental evidences above illustrate that AaMYB15 is a negative regulator of artemisinin biosynthesis in *A. annua.* Overexpression of *AaMYB15* led to decline of expression levels of *ADS, CYP71AV1, DBR2,* and *ALDH1*, thereby causing a significant decline in artemisinin content, while repressing the expression of *AaMYB15* brought the opposite consequence.

### 3.5 AaMYB15 inhibits the promoter activities of *ADS*, *CYP71AV1*, *DBR2*, and *ALDH1*

**Fig. 5:**
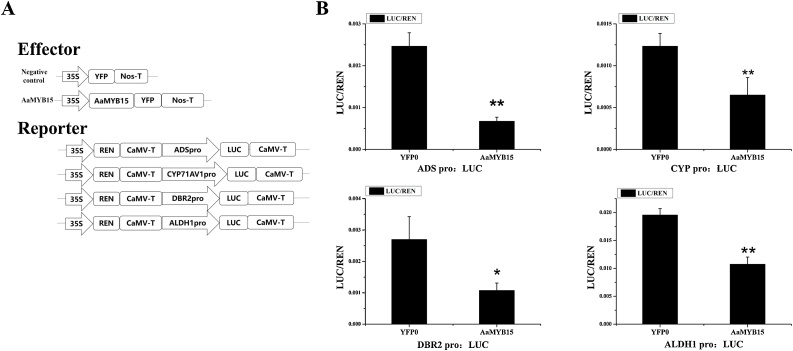
AaMYB15 inhibits promoter activities of *ADS*, *CYP71AV1*, *DBR2* and *ALDH1.* (A) Carriers construction diagram of the reporters and effectors used in the dual-luciferase assay. (B) Inhibition of *ADS* (a), *CYP71AV1* (b), *DBR2* (c) and *ALDH1* (d) gene promoter activities by AaMYB15 in dual-luciferase assay. The strengths of activities are represented by the LUC/REN ratio. Asterisks indicate significant differences between AaMYB15 and the negative control by Student’s *t*-test. (**, P < 0.01；*,P < 0.05.)Values are given as means ± SD from three biological replicates.

For the purpose of studying the specific mechanism of AaMYB15 regulating artemisinin biosynthesis ulteriorly, Dual-Luciferase assays were performed to scrutinize the relationship between AaMYB15 and the promoter activities of key enzymes genes *ADS, CYP71AV1, DBR2, and ALDH1* (Fig. 5, A). Promoter activities were evaluated with the effector AaMYB15 and empty vector as a negative control. As shown in figure (Fig. 5, B), when AaMYB15 was co-transformed with *ADSpro*: LUC, *CYP71AV1pro*: LUC, *DBR2pro*: LUC, and *ALDH1pro*: LUC, promoter activities were significantly decreased in comparison with negative control. These results marked that AaMYB15 repressed the promoter activities of *ADS, CYP71AV1, DBR2,* and *ALDH1* and acted as a negative regulator of artemisinin biosynthesis.

### 3.6 AaMYB15 directly binds to the promoter of *AaORA* and suppresses its activity

As demonstrated by results in preceding paragraphs, AaMYB15 is proved to be a negative regulator of artemisinin biosynthesis and suppresses transcriptional activities of key enzymes genes. Nevertheless, direct binding of AaMYB15 to key enzyme genes promoters was not detected by yeast one-hybrid assays (Fig. S2), implying that there were other transcription factors functioning downstream AaMYB15 to participate in regulating expression of key enzymes.

To find the downstream regulators of AaMYB15, Y1H and Y2H vectors of multiple reported positive regulators involved in light or JA signaling pathways were constructed to detect whether there were interactions between proteins by Y2H (including AaGSW1, AaHD1, AaERF1, AaJAZ8, AabHLH1, AabZIP1, AaHY5, AaCOP1)or between proteins and whole promoters by Y1H (including *proAaORA*, *proAaMYC2*, *proAaGSW1*) with AaMYB15 [21,43]. (Figs. S2 & S3), but no interaction was observed.

Previous studies have implied that R2R3-MYB TFs recognize binding sites rich in A, C base [44], and abounding MYB binding sites (MBS) on DNA have been reported. Analysis of promoters by PlantCARE (http://bioinformatics.psb.ugent.be/webtools/plantcare/html/) predicted numerous MYB binding sites in the promoter region of those genes mentioned above.

**Fig. 6:**
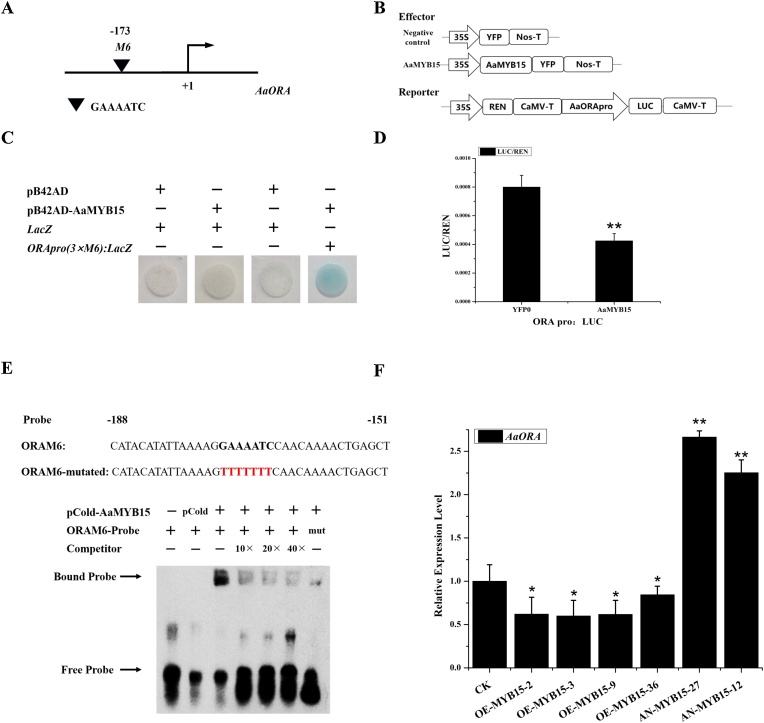
AaMYB15 directly binds to the promoter of *AaORA* and suppresses its activity. (A) Schematic diagrams of *AaORA* promoter. The position of potential MYB DNA binding site M6 is shown as black triangle and numbered on the basis of the distance from the translational start site (ATG) set as +1. (B) Carriers construction diagram of the reporters and effectors used in the dual-luciferase assay. (C) Yeast one-hybrid assay showing that AaMYB15 binds to the predicted MYB binding site M6 in *AaORA* promoter. Three tandem repeats of the motif were used as baits. Yeast cells co-expressing relevant plasmids were grown on selective medium, SD/-Trp/-Ura, containing X-gal (20 mg/liter), and pictures were taken after three days of incubation at 30 °C. Blue plaque indicates protein-DNA interactions. The assay was repeated three times. (D) Inhibition of *AaORA* promoter activities by AaMYB15 in dual-luciferase assay. The strengths of activities are represented by the LUC/REN ratio. Asterisks indicate significant differences between AaMYB15 and the negative control by Student’s *t*-test. (**, P < 0.01；*,P < 0.05.)Values are given as means ± SD from three biological replicates. (E) EMSA showing that AaMYB15 binds to the predicted MYB binding site M6 in *AaORA* promoter. pCold -TF protein was used as negative control and unlabeled probes as competitor. (F) Expression levels of *AaORA* in overexpression of *AaMYB15*(OE-MYB15) and antisense-*AaMYB15*(AN-MYB15) transgenic A. annua. Values are given as means ± SD from three biological replicates. Asterisks indicate significant differences between transgenic plants and CK by Student’s *t*-test. (**, P < 0.01；*,P < 0.05.)

Y1H assay and EMSA exhibited that AaMYB15 bound to the sixth MYB binding motif, tentatively named *M6* (173bp upstream of ATG, respectively) in *AaORA* promoter region, thus activating the expression of the *LacZ* reporter gene (Fig. 6, C& E), revealing that AaMYB15 directly bound to the promoter of *AaORA*. Afterwards, it was found by Dual-luciferase assay that the LUC/REN ratio was evidently decreased when pGREENII0800-AaORA-LUC was co-transformed with AaMYB15 (Fig. 6, D) in contrast to negative control, purporting that AaMYB15 inhibited the promoter activity of *AaORA*. Expression levels of *AaORA* in OE-MYB15 and AN-MYB15 transgenic *A. annua.* seedlings were detected by qRT-PCR likewise (Fig. 6, F), demonstrating that transcriptional activity of *AaORA* was restrained in OE-MYB15 plants and obviously upregulated in AN-MYB15 plants.

Considering that AaORA was affirmed to upregulate expression levels of multiple key enzyme genes, these results indicated that AaMYB15 directly bound to the promoter of *AaORA* and suppressed its activity, accordingly down-regulated expression levels of key enzymes genes and reduced the artemisinin content in *A. annua*, as well.

## 4 Discussion

Previous studies have elucidated that ADS, CYP71AV1, DBR2, and ALDH1 are four key rate-limiting enzymes in downstream pathway of artemisinin biosynthesis [[12], [13], [14], [15]] and GSTs are the locations of artemisinin biosynthesis and storage where genes related to artemisinin biosynthesis are specifically or dominantly expressed [[45], [46], [47]]. Meanwhile, the biosynthesis of artemisinin is also affected by many environmental factors, including phytohormones [48], stress factors [49] and biotic, chemical or physical factors [[33], [34], [35],[50], [51], [52], [53]]. Light signaling has been reported to be a crucial positive environmental regulatory factor in artemisinin biosynthesis. Meanwhile, JA is also described as a most important phytohormone to regulate artemisinin biosynthesis, and it is reported that the JA-mediated regulation is dependent on light [36].

As essential tools in plant metabolic engineering, numbers of transcriptional factors have been identified, to regulate the expression of artemisinin biosynthesis genes or the density of GSTs on the surface of *A. annua,* which ultimately affect the synthesis of artemisinin through various signal pathways [54].Most of these studies are about positive regulators, while little attention is paid to the negative regulators.

Nevertheless, with a view to negative regulators, researchers can obtain instructive evidences to analyze various regulatory networks more thoroughly and gain deeper understanding of the mechanism of dynamic balance which extensively functions in secondary metabolism pathways. Taking this as a starting point, combining transcriptome sequencing data of our previous study, we expected to identify a negative regulator of artemisinin biosynthesis in light and JA signaling pathways.

In this study, an R2R3- MYB TF AaMYB15 was previously discovered as a DEG from transcriptome analysis to light, dark and JA treatment. Consistent with the precondition, AaMYB15 was found induced by dark and JA treatment and repressed in light condition by qRT-PCR analysis (Fig. 3). Taken together with the subcellular localization in nucleus (Fig. 2, A), spatial and temporal expression patterns (Fig. 2, C and D) and expressed pattern in GSTs presented by GUS staining (Fig. 2, B), AaMYB15 was envisioned to be a potential negative regulator of artemisinin biosynthesis in regulatory pathways mediated by light and JA. QRT-PCR analyses of *AaMYB15* overexpression and antisense transgenic plants showed decreased expression levels of *ADS, CYP71AV1, DBR2,* and *ALDH1* and marked decline of artemisinin contents in OE-*AaMYB15* lines, demonstrating that AaMYB15 downregulated key enzyme genes and artemisinin biosynthesis in *A. annua* (Fig. 4, B and C). Then, Dual-Luciferase assay (Fig. 5) revealed that AaMYB15 inhibited the promoter activities of *ADS, CYP71AV1, DBR2,* and *ALDH1*. However, there was no evidence supporting that AaMYB15 directly regulated expression of key enzymes genes, suggesting the presence of other parallel or downstream regulators.

AaORA is a valuable AP2/ERF TF reported to positively regulate *ADS, CYP71AV1,* and *DBR2* genes directly or associated with other ERF TFs [37] and AaTCP14 [21]. AaMYB15 was proved to directly bind the MYB binding site *M6* (Fig. 6, A) in promoter of *AaORA* by Y1H (Fig. 6, C) and suppressed the transcriptional activity of *AaORA* in *N. benthamiana* (Fig. 6, D) and *A. annua* (Fig. 6, E), thus downregulating the artemisinin biosynthesis. The mechanism of the function of AaMYB15 was further clarified.

On the other hand, there are still questions about AaMYB15 worth studying in depth. AaORA is reported to positively regulate *ADS*, *CYP71AV1*, and *DBR2*. Since its regulatory effect on *ALDH1* is not observed, the way AaMYB15 downregulates *ALDH1* is still unexplained, remains to be unceasingly explored. The relationship between AaMYB15 and other central regulators in light or JA-mediated regulation is also need to be investigated to ulteriorly figure out the role it plays in these signaling pathways. Besides, light and JA signaling are generally recognized as positive influence factors of artemisinin biosynthesis, but AaMYB15 shows a light-inhibited and JA-induced expression pattern. This phenomenon confirms the complexity of transcriptional regulation of artemisinin biosynthesis, which is closely related to dynamic balance between transcription promotion, inhibition and between external environment and transcription factor regulatory network.

In summary, AaMYB15, the first negative R2R3-MYB regulator of artemisinin biosynthesis involved in light and JA signaling pathways from *A. annua*, was identified and functionally characterized in this study. Our study provides AaMYB15 as a candidate gene in genetic engineering for cultivation of *A. annua* lines with high artemisinin yield and genetic improvement of *A. annua* germplasm. Moreover, this study brings implication for future research on the overall network of regulation mediated by light and JA on artemisinin biosynthesis in *A. annua* and contributes to unveil the mystery of the dynamic balance mechanism.

## Declaration of Competing Interest

The authors report no declarations of interest.

## Figures and Tables

**Figure:**
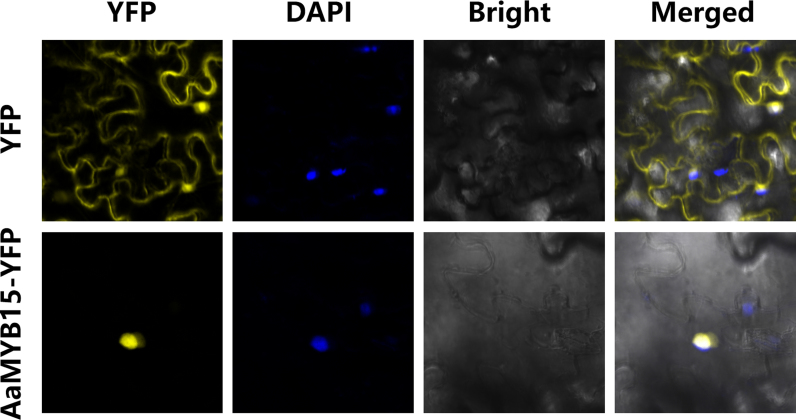


**Figure:**
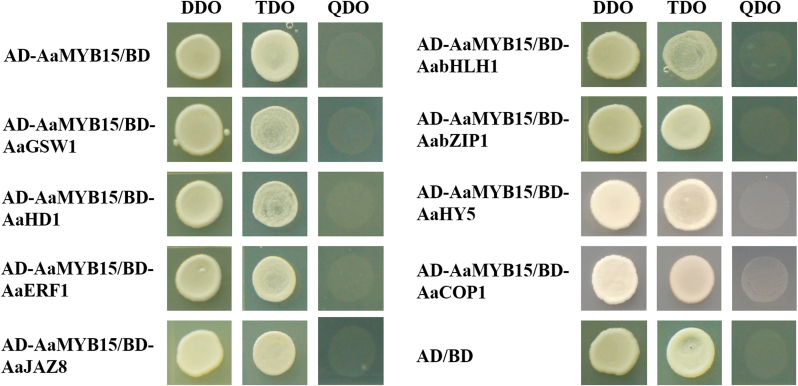


**Figure:**