# Supplementary material for: AaMYB15, an R2R3-MYB TF in Artemisia annua, acts as a negative regulator of artemisinin biosynthesis
Source: Plant Sci. 2021 Jul;308:None. doi: 10.1016/j.plantsci.2021.110920 (PMC8174473; doi:10.1016/j.plantsci.2021.110920)
Supplement: Supplementary file 1 [file mmc1.docx]

**Supplemental Information**

Table. S1 Primers and probes mentioned in this article

Fig. S1 Subcellular localization assays with DAPI used to stain nuclei

Fig. S2 Y2H assays between AaMYB15 and some regulators involved in light or JA signaling pathways

Fig. S3 Y1H assays between AaMYB15 and whole promoters of four key enzymes and *proAaORA*, *proAaMYC2*, *proAaGSW1*
